# Micromorphology of the Adhesive Interface of Self-Adhesive Resin Cements to Enamel and Dentin

**DOI:** 10.3390/ma14030492

**Published:** 2021-01-20

**Authors:** Konstantin Johannes Scholz, Aleksandra Bittner, Fabian Cieplik, Karl-Anton Hiller, Gottfried Schmalz, Wolfgang Buchalla, Marianne Federlin

**Affiliations:** 1 Department of Conservative Dentistry and Periodontology, University Hospital Regensburg, Franz-Josef-Strauß-Allee 11, 93053 Regensburg, Germany; Aleksandra.bittner@googlemail.com (A.B.); fabian.cieplik@ukr.de (F.C.); Karl-anton.hiller@ukr.de (K.-A.H.); Gottfried.schmalz@ukr.de (G.S.); Wolfgang.buchalla@ukr.de (W.B.); marianne.federlin@ukr.de (M.F.); 2 Private Practice, 01067 Dresden, Germany; 3 Department of Periodontology, Center of Dental Medicine, University of Bern, 3012 Bern, Switzerland

**Keywords:** dental bonding, scanning electron microscopy, self-adhesive resin cements, SEM evaluation

## Abstract

Interfaces between dentin, enamel and luting agents were characterized using low vacuum Scanning Electron Microscopy (SEM). After smear layer creation, one of three luting agents (RelyX Unicem 2, Clearfil SA Cement and Panavia F 2.0/ED Primer II) was applied on 60 enamel-dentin specimens and dual-cured or self-cured. Specimens were polished (Experiment 1) and subsequently demineralized and deproteinized (Experiment 2). Adhesive interfaces were analyzed (low vacuum SEM, ×3000). Presence of an interdiffusion zone, tag-like structures (dentin) and marginal gaps (enamel) were assessed. Non-parametrical tests (χ^2^-test, α = 0.05) were performed. The first null-hypothesis was that the adhesive interface micromorphology between enamel and dentin and self-adhesive resin cements (SARCs) is similar with conventional resin cement used with a self-etch adhesive (CRC+SE). The second null-hypothesis was that the micromorphology is not influenced by curing modes. Interdiffusion zones and tag-like structures (dentin) were observed more frequently for CRC+SE compared to SARCs. For each luting agent, there was a non-significant (*p* > 0.05) tendency for interdiffusion zone and tag-like structures detectable in more specimens after self-curing compared to dual-curing. Marginal gaps (enamel) were found only for SARCs. The first null-hypothesis was not rejected fully: Tag-like structures and interdiffusion zones in dentin were found for CRC+SE and SARCs. The second null-hypothesis was not rejected.

## 1. Introduction

A sufficient and clinically reliable bond between dental tissues and restorations is a crucial precondition for resin bonded partial crowns, crowns, and bridges [1]. Self-adhesive resin cements (SARCs) have shown promising clinical outcomes resulting in a survival rate of 97.6% over 6 years for metal-based crowns, a survival rate of 83.5% for lithium disilicate crowns over 10 years and survival rates of above 90% for zirconia fixed dental prostheses over 6 years [2,3,4]. SARCs are designed to combine the ease of use of glass ionomer cements and the favorable mechanical and adhesive properties of conventional resin cements in combination with a beforehand applied adhesive system (CRC+SE) [5]. While conventional resin cements like Panavia F 2.0 (Kuraray, Tokyo, Japan) require pretreatment of the tooth surface with acidic primers or self-etch adhesives, SARCs comprise acid-functionalized monomers that aim to directly demineralize and chemically interact with dental tissues by formation of calcium salts [6]. Although conventional resin cements show better bond strength values in vitro compared to SARCs [7], the higher number of working steps for conventional resin cements may be disadvantageous in clinical situations.

Following preparation using rotating instruments during restorative procedures, human dentin is typically covered with an up to 5 µm thick acid-soluble smear layer consisting of ground organic and inorganic dentin components [8,9]. A smear layer also forms on enamel, but with a thickness below 1 µm [10]. The smear layer thickness depends on the instruments used for preparation, with more coarse diamond burs leading to a thicker dentin smear layer [10,11]. In order to obtain a stable bond between dentin and the SARC, interaction with the smear layer is crucial, particularly for SARCs, which can be used clinically without etch-and-rinse pretreatment of the tooth surface [12]. A smear-layer with reduced thickness as produced by finishing instruments may lead to a higher dentin bond strength for mild self-etch adhesives or self-adhesive restorative materials compared to thicker smear-layers as produced by carbide-burs [13,14,15,16].

Differences in luting agent composition may affect the adhesive interface to dentine and enamel. However, information on and particularly visualization of these interfaces is hardly available. The aim of this study was to enroll low vacuum scanning electron microscopy (LV-SEM) under low voltage conditions to visualize the micromorphology of the adhesive interfaces between human enamel and dentin, and three different luting materials: two SARCs used without a separately applied self-etch adhesive and one CRC+SE. Each luting agent was applied in both, dual-curing and self-curing mode. Micromorphological visualization of the adhesive interfaces was performed by means of LV-SEM under low voltage conditions below 5 kV, which offers visualization of specimens with increased sensitivity to the chemical nature and topography of the specimen surface and reduced specimen charging [17,18]. To the best knowledge of the authors, there are no studies investigating SARCs under low voltage conditions in LV-SEM.

The null-hypothesis was that micromorphological interactions between tooth substances and restorations are not different for the luting agents tested. In detail, the first null-hypothesis was that micromorphological interactions with enamel (tight marginal seal) or dentin (interdiffusion zones and tag-like structures) found with CRC+SE are also present in the adhesive interface with SARCs. The second null-hypothesis was that the micromorphology is not influenced by dual-curing (light- and self-curing combined) or self-curing mode.

## 2. Materials and Methods

### 2.1. Specimen Selection

**Figure 1:**
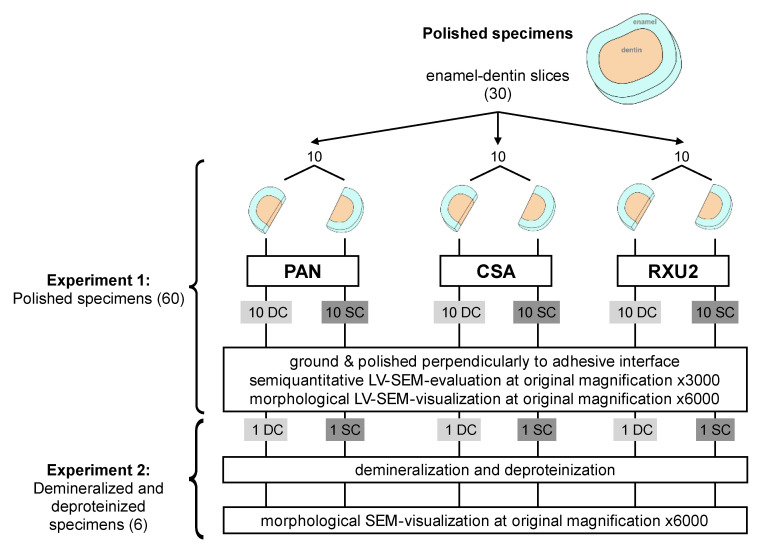
Allocation of enamel dentin slices for experiment 1 (polished specimens) and experiment 2 (demineralized and deproteinized specimens). For Panavia F 2.0 (PAN), Clearfil SA Cement (CSA) and RelyX Unicem 2 (RXU2), the two semicircular specimens arising from the same tooth were allocated to dual-curing mode (DC) and self-curing mode (SC). Specimens were evaluated and visualized using low vaccum scanning electron microscopy (LV-SEM) under low voltage conditions.

The use of human teeth, with informed consent from each donor, was approved by the internal review board of the University of Regensburg (ref. 19-1327-101). Thirty-six extracted caries-free human third molars were cleaned from all residual external soft tissues and stored in 0.5% chloramine T trihydrate solution (Merck KGaA, Darmstadt, Germany) for a maximum of 1 month at 4 °C prior to use. Six specimens were used for measurement of smear layer thickness. Thirty specimens were used for semiquantitative analyses of the polished interfaces (Experiment 1), six out of them were used further for morphological visualization of the polished interfaces after demineralization and deproteinization (Experiment 2, Figure 1). 

### 2.2. Smear Layer Creation and Measurement on Fractured Specimens

One 2 mm thick exactly plane-parallel enamel-dentin slice was cut horizontally from the mid-coronal region of 6 third molars (Innenlochsäge Leitz 1600, Leica Mikrosysteme Vertrieb GmbH, Wetzlar, Germany; blade thickness 300 µm) using copious water cooling. A central predetermined breaking line was notched centrally into the pulpal surface of the 6 enamel-dentin slices in bucco-lingual direction using a water-cooled diamond disc (Superdiaflex H 365F 190, Horico Dental, Berlin, Germany). A standard smear layer was produced as follows: The coronal surfaces of each of these 6 specimens were ground wet by one operator (A.B.) for 60 s using a circular motion and finger pressure on the sandpaper lying on a laboratory bench (600 grit ANSI/CAMI; Carbimet Paper Discs, Buehler, Lake Bluff, IL, USA), rinsed with demineralized water (1.82 × 10^7^ µSv; TKA GenPure, TKA xCAD, TKA Wasseraufbereitungssysteme GmbH, Niederelbert, Germany), and gently dried with compressed air up to the point that all visible water was removed. Subsequently, the specimens were fractured by hand along the predetermined breaking line to expose a perpendicular view onto the undisturbed dentin covered with a smear layer and smear plugs. 

### 2.3. LV-SEM Smear Layer Examination of Fractured Specimens

The specimens were mounted onto aluminum stubs (Baltic Präparation, e.K., Wetter, Germany) using double-sided adhesive carbon discs and conductive adhesive paste (Leit-C-Plast and Leit-C-Tab, Plano GmbH, Wetzlar, Germany). SEM-micrographs of the undisturbed and uncovered smear layer were taken vertically from the fractured surface of each specimen in low vacuum mode within 4 h of preparation for SEM evaluation without sputtering (FEI Quanta 400 FEG, Thermo Fisher Scientific, FEI Deutschland GmbH, Frankfurt a. M., Germany; secondary electron mode, 1.5 Torr, accelerating voltage 4 kV, WD 6.5 mm, spot size 4.0, pressure limiting aperture 500 µm, image resolution 2048 × 1768 pixels, horizontal field width 90.13 µm at ×3000 original magnification with a resolution of 45 nm). The smear-layer thickness on dentin was measured on the 6 fractured specimens at original magnification ×6000 at 5 randomly chosen locations in a distance of 10 µm from one another. 

### 2.4. Luting Agents and Curing Modes

A SARC containing monomers with two phosphoric and two polymerizable groups (RXU2; RelyX Unicem 2, color A2, 3M Oral Care, Seefeld, Germany) and a SARC containing monomers with one phosphoric and one polymerizable group (CSA; Clearfil SA Cement, color A2, Kuraray, Tokyo, Japan) were used in this study. A 10-MDP (10-methacryloyloxydecyl dihydrogen phosphate)-containing CRC+SE (PAN; Panavia F 2.0, shade TC, ED Primer II, Kuraray, Tokyo, Japan) served as control. For better readability, the term luting agents is used throughout this manuscript for both, SARCs and CRC+SE. The composition of each luting agent as specified by the manufacturer is shown in Table 1 [19]. All luting agents were used in dual-curing (DC) comprising light-curing and a subsequent dark-curing period, and self-curing (SC) mode comprising dark-curing only.

#### Experiment 1: Semiquantitative Analyses of the Polished Interface 

**Figure 2:**
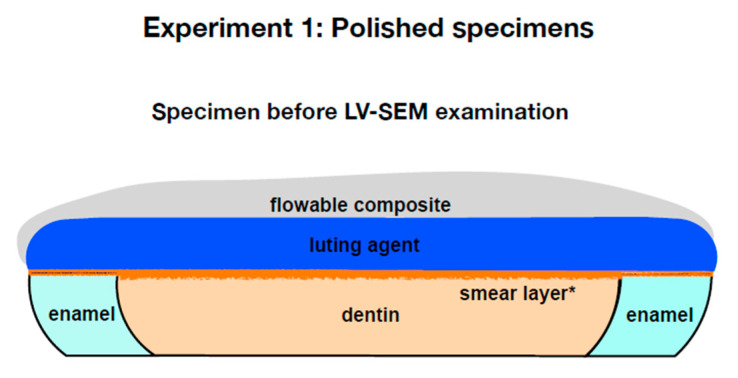
Polished specimens after preparation and before low vacuum scanning electron microscopy (LV-SEM) (experiment 1). For dual-curing mode (DC), specimens were polymerized for 60 s. A vertical limit-stop was used in order to better align the components of the specimens. For self-curing mode (SC), specimens were allowed to polymerize for 24 h isolated from light. * = smear layer was created by wet grinding.

**Figure 3:**
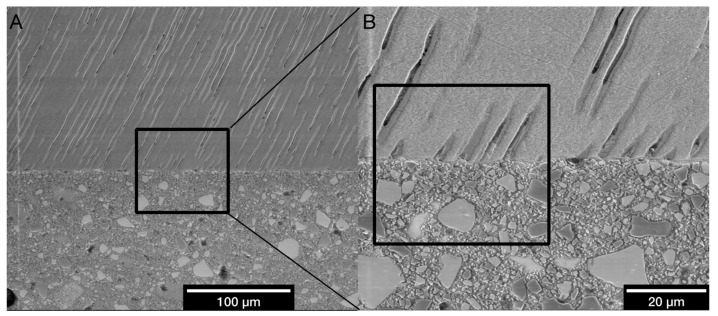
Exemplary area selection (PAN, DC) for semiquantitative analysis. (**A)** Adhesive interface in lateral dentin at ×800 original magnification. Black square: Area for the micrograph at ×3000 original magnification. (**B**) Adhesive interface in lateral dentin at ×3000 original magnification for semiquantitative analysis. Black square: Area for the micrograph at ×6000 original magnification for exemplary descriptive depiction.

Thirty 2 mm thick enamel-dentin slices were prepared from human third molars as described above and cut through the center in bucco-lingual direction into two equal-sized specimens using a water-cooled diamond disc (Superdiaflex H 365F 190, Horico Dental, Berlin, Germany) resulting in 60 semicircular shaped enamel-dentin specimens (Figure 1, Figure 2 and Figure 3). A smear layer was created on the coronal surface of the semicircular specimens as described above. A vertical limit-stop was placed against the cut surface in order to better align the components of the specimens and to avoid surplus luting agent. The two halves of an enamel-dentin slice were treated with one luting agent, one half in dual-cure mode (DC), the other in self-cure mode (SC). Specimens were allocated to experimental groups as shown in Figure 1.

All luting agents were applied by the same dentist. The coronal surface was not treated with an additional adhesive step before application of the two SARCs RelyX Unicem 2 and Clearfil SA Cement using an application tool (Applicator gun for cement capsules, 3M Oral Care, Seefeld, Germany) according to the manufacturer instructions. Before application of Panavia F 2.0, ED Primer II was carefully applied according to the manufacturer’s guidelines (mixing of components A and B, application, 30 s exposure without agitation, gentle air-drying). Panavia F 2.0 was mixed and applied on the surface as stated by the manufacturer (mixing of Paste A and Paste B by hand for 20 s). The coronal surface of each specimen was covered with the respective luting agent approximately 1 mm thick and covered by a clear polyester strip (Universal strips, Frasaco, Tettnang, Germany). In accordance with a common thickness of ceramic restorations in a clinical situation, a CAD/CAM-fabricated (computer aided design/computer aided manufacturing) semicircular shaped ceramic slice of 2 mm thickness (VITA Mark II 2M2C, VITA Zahnfabrik, Bad Säckingen, Germany) was placed on top and loaded with 420 g weight. Hereby excess cement was extruded and removed. One half of each original dentin-enamel slice that was assigned to the dual-cure group (DC) was immediately light-cured for 60 s using an LED curing light (Bluephase C8, Ivoclar Vivadent, Schaan, Liechtenstein; light intensity ≥ 945 mW/cm^2^ according to a Cure Rite Visible Curing Light Meter, Dentsply Caulk, Milford, CT, USA) perpendicularly to the accessible adhesive interface and through the ceramic disk at 5 mm working distance in accordance with the working distance in a clinical situation, when posterior teeth are restored. The other half (group SC) was stored in a dark incubator (U-10, Memmert, Schwabach, Germany) in 100% humidity at 37 °C for 10 min to allow self-curing. After the polymerization phase, the ceramic slice and the clear strip was removed from the specimens from both curing groups. The specimens were stored in an incubator (U-10, Memmert, Schwabach, Germany) in 100% humidity at 37 °C for another 24 h. Subsequently, the luting agents were reinforced with a 1 mm layer of flowable composite (Tetric EvoFlow A2, Ivoclar Vivadent, Schaan, Liechtenstein) that was light-cured for 60 s (Figure 2).

Overall, 20 specimens from 10 different enamel-dentin slices were allocated to each luting agent. Ten of these were dual-cured (light- and self-curing combined) and 10 self-cured (Figure 1). The specimens were wet ground and polished perpendicularly to the tooth-cement interface from the flat side by hand (600, 800, and 1200 ANSI/CAMI grit; Carbimet Paper Discs, Buehler, Lake Bluff, IL, USA) for 1 min per step with 1 min rinsing with water spray in between steps) with a carefully applied uniform pressure. The surface was evenly moved by one operator (A.B.) over the sandpaper lying flat on a laboratory bench with finger pressure. By doing this, the flatness and parallelism of the specimens was not compromised prior to adhesive application. Consecutively, the specimens were hand-polished with alumina-slurry (1 min per step, 1 min rinsing with water spray in between steps; Mastertex PSA 8”, alumina suspension of 1.0–0.05 µm grain size; Buehler, Lake Bluff, IL, USA).

### 2.5. LV-SEM of Polished Specimens (Experiment 1)

Following preparation for LV-SEM examination as described above for fractured specimens, the polished surface of each specimen that had been placed against a vertical limit-stop before was examined using the same LV-SEM parameters as described above. Micrographs were recorded at original magnifications of ×800, ×3000, and ×6000 in enamel, central, and lateral dentin, resulting in 9 micrographs per specimen. Micrographs at an original magnification of ×800 were used as an overview, micrographs at an original magnification of ×3000 were evaluated semiquantitatively, and micrographs at an original magnification of ×6000 were taken to exemplarily depict the specific characteristics of the adhesive interface (Figure 3). Aiming for a semiquantitative, mainly descriptive evaluation of the adhesive interfaces, the presence or absence in the respective specimen of the following morphological criteria for characterization of the adhesive interfaces was used for semiquantitative evaluation: interdiffusion zone, tag-like-structures in dentin, and marginal gaps in enamel. 

The criteria interdiffusion zone and tag-like structures were evaluated in dentin. The interdiffusion zone was defined as a morphologically visible zone of interaction between dentin and luting agent of variable thickness [20]. The interdiffusion zone—similar to the hybrid layer after acid-etching and adhesive infiltration—may represent the smear layer or demineralized and hybridized dentin modified by interaction with the materials used. Tag-like structures are formed by luting agent or components thereof infiltrating partially or completely open dentinal tubules near the interface between luting agent and dentin [21,22]. With respect to enamel, marginal gaps were defined as loss of a tight adhesion between enamel and luting agent [21].

Specimens with presence or absence of each morphological criterion were counted. The presence or absence of respective criteria was evaluated in three micrographs per specimen: one for enamel halfway between the outer tooth surface and the dentino-enamel junction, one for central dentin, and one for lateral dentin next to the dentino-enamel junction (DEJ) at an original magnification of ×3000. The criteria interdiffusion zone and tag-like structures were rated as present in the respective specimen, if they were found in at least one of the two evaluated micrographs (central or lateral dentin). Marginal gaps were rated as present if detected partially or completely in the evaluated micrograph in enamel.

#### Experiment 2: Morphological Analysis of the Polished Interface after Demineralization and Deproteinization

This newly introduced LV-SEM method under low voltage conditions for depiction of the interdiffusion zone on polished specimens was compared to a commonly used protocol involving the removal of the dentin and exposure of the interface morphology. One specimen showing a characteristic interface morphology was further processed by demineralization (15 s HCl 1 N, Merck KGaA, Darmstadt, Germany) and deproteinization (10 min, 2% NaOCl, Speiko, Münster, Germany) at the cross-sectioned polished area [23].

### 2.6. LV-SEM of Demineralized and Deproteinized Specimens (Experiment 2)

The demineralized and deproteinized specimens were examined again at the same sites and magnifications as before demineralization and deproteinization using the same LV-SEM parameters as described above.

### 2.7. Data Analysis

Considering measurement of smear layer thickness, the median of the single measurements at 5 locations was used as the representative value of each specimen. Median, 25%- and 75%-percentiles from specimens’ representative values were determined. Considering experiment 1, qualitative micromorphological data from SEM examinations for each group in dentin (n = 10) and in enamel (n = 10) were expressed as frequencies and percentages based on all specimens that were not excluded from evaluation due to fractures or total loss of adhesion due to preparation. χ^2^ tests were performed at an α = 0.05 level of significance to examine whether curing modes and luting agents were correlated with respect to interdiffusion zone, tag-like structures and marginal gaps. Considering experiment 2, the aim was to visualize the interface between the tooth structure and the restoration on a single specimen. These morphological images were not subjected to statistical analysis. All data were analyzed using SPSS, version 25 (SPSS Inc., Chicago, IL, USA).

## 3. Results

### 3.1. Measurement of Smear Layer Thickness Using Fractured Specimens

**Figure 4:**
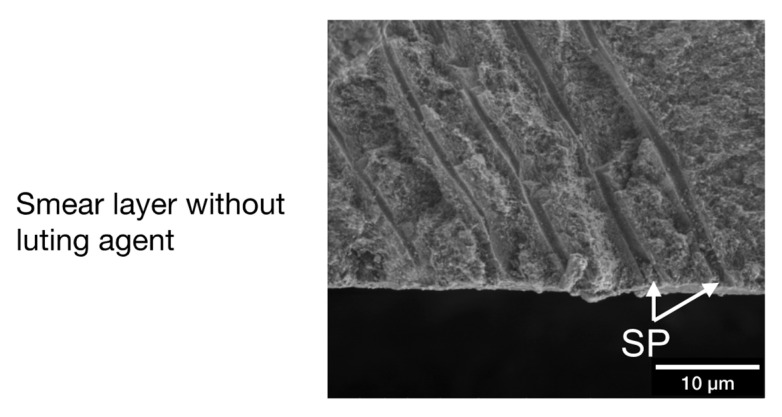
Exemplary micromorphology of a fractured specimen without material application revealing an undisturbed smear layer with smear plugs (original magnification ×6000).

An undisturbed smear layer including smear-plugs in the orifices of dentinal tubules was detectable in all fractured specimens (Figure 4) showing a median (25% percentile; 75% percentile) smear layer thickness of 620 (550; 721) nm. Smear plugs occurring at the orifices of dentinal tubules were visible in fractured specimens. Smear plugs had a more irregular appearance, were limited to the entrance of dentinal tubules, and revealed a rougher and more porous surface compared to tag-like structures.

### 3.2. Micromorphological Interactions

#### 3.2.1. Experiment 1: Semiquantitative Evaluation of Polished Specimens

**Figure 5:**
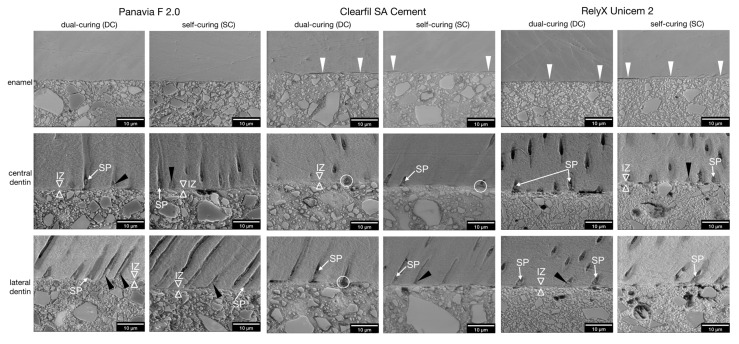
Adhesive interface micromorphology in polished specimens (Experiment 1) after dual-curing or self-curing (original magnification ×6000). Left panel: Marginal gaps between enamel and luting agent could not be observed in any of the PAN specimens. No deeper interaction or etching pattern could be revealed in enamel. In dentin, a distinct interdiffusion zone (IZ) was visible in half of all specimens. Smear plugs (SP) and broad tag-like structures (black arrow heads) were present inside the dentinal tubules. To demonstrate the are selection, the respective micrographs of PAN, DC, lateral dentin at ×800 and ×3000 original magnification are shown in Figure 3. Middle panel: In CSA specimens, marginal gaps (white arrow heads) are visible for DC and SC between enamel and luting agent, which were interrupted by areas with intact bond. In dentin, a thin interdiffusion zone of approximately 1 µm (IZ) is barely visible. Typically, pores in the luting agent were located near the orifices of dentinal tubules (white circles), possibly representing water-inclusion during material setting. Smear plugs (SP, white arrows) and tag-like structures (black arrow heads) were detected. Right panel: In RXU2 specimens, marginal gaps (white arrow heads) were found for DC and SC at the interface as well as cohesive fractures within the enamel. In dentin, smear plugs (SP) and tag-like structures (black arrow heads) representing infiltration of resin-matrix with and without filler particles into dentinal tubules coexisted. Thin interdiffusion zones (IZ, hollow white arrow heads; ~1 µm) could be detected.

**Figure 6:**
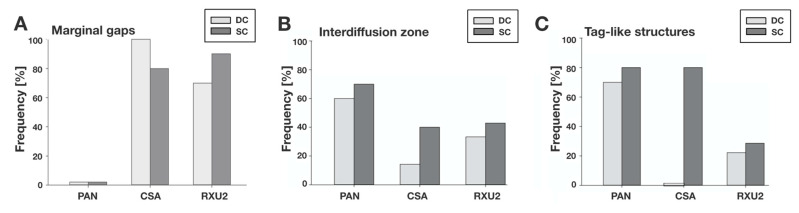
Frequency (percent of specimens with the respective findings of the adhesive interface micromorphology) by luting agent, after dual-curing (DC) or self-curing (SC) based on 10 polished specimens per group (experiment 1). (**A**) Marginal gaps between enamel and luting agents (n = 10). (**B**) Interdiffusion zones from the central or lateral dentin area were evaluated in each of the 10 specimens per group. The criterion was rated as present in the respective specimen if it was found in at least one of the two evaluated micrographs (central or lateral dentin). (**C**) Tag-like structures from the central or lateral dentin area were evaluated in each of the 10 specimens per group. The criterion was rated as present in the respective specimen if it was found in at least one of the two evaluated micrographs (central or lateral dentin).

In enamel, the examination of all 10 polished specimens per group allowed an evaluation of the presence of marginal gaps on the interface between enamel and resin at an original magnification of ×3000 (Figure 5 and Figure 6).

For PAN, no marginal gaps were detected in either DC or SC mode in any specimens (Figure 5 and Figure 6). For CSA, marginal gaps were detected in 100% of the specimens after DC and 80% after SC (Figure 5 and Figure 6). For RXU2, marginal gaps were detected in 70% of the specimens after DC and 90% after SC (Figure 5 and Figure 6). No other surface irregularities of enamel were detected for any of the materials or curing modes. The loss of the adhesive bond occurred on localized areas of the adhesive interface between luting agent and enamel in all specimens from both SARCs, but no total loss of the adhesive bond between luting agent and enamel was recorded. No significant differences of the presence of marginal gaps between DC and SC could be detected for any luting agent (*p* > 0.05).

In dentin, specimens showing fractures or total adhesion loss in central and lateral dentin due to specimen preparation were excluded from micromorphological evaluation at ×3000 original magnification. Thus, the values for interdiffusion zone and tag-like structures resulted from 20 specimens for PAN (10 DC, 10 SC), 12 specimens for CSA (7 DC, 5 SC), and 16 specimens for RXU2 (9 DC, 7 SC) (Figure 6). Missing tag-like structures near the interface were observed more often with CSA and RXU2 than compared to PAN (Figure 5).

For PAN, an interdiffusion zone was detectable in 60% of the specimens after DC and in 70% after SC. Tag-like structures were detected in 70% in DC mode and 80% in SC mode. For CSA, an interdiffusion zone was found in 14% after DC and 40% after SC. Tag-like structures were detected in 0% in DC mode and in 80% in SC mode. For RXU2, an interdiffusion zone was detectable in 33% after DC and 43% after SC. Tag-like structures were detected in 22% after DC and 29% after SC.

For all luting agents, there was a tendency for interdiffusion zone and tag-like structures to be found in more specimens after SC compared to DC although this was not statistically significant (*p* > 0.05).

#### 3.2.2. Experiment 2: Demineralized and Deproteinized Specimens, Morphological Characteristics

**Figure 7:**
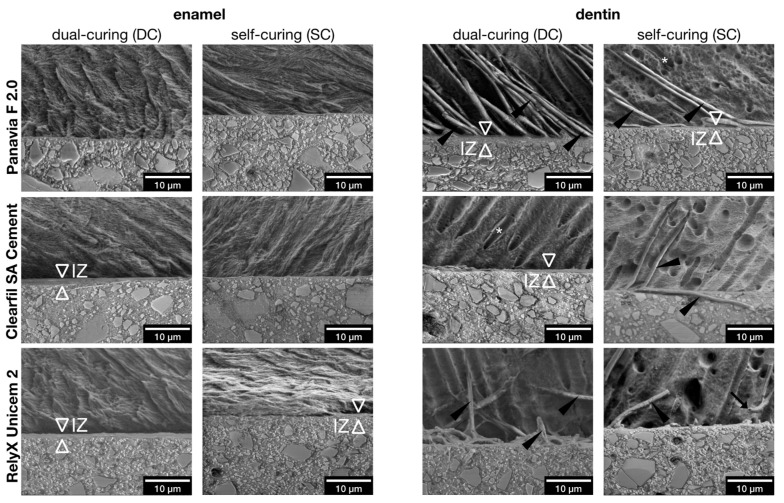
Adhesive interface micromorphology in polished specimens after demineralization and deproteinization (experiment 2; original magnification ×6000). Left panel: Adhesive interface micromorphology in specimens from all luting agents with enamel in DC and SC mode after demineralization and deproteinization. Thin interdiffusion zones (IZ) were visible. Right panel: Adhesive interface micromorphology in specimens from all luting agents with dentin in DC and SC mode after demineralization and deproteinization. Interdiffusion zones (IZ), tag-like structures (black arrow heads) and tags with inner hollow spaces (black arrows) and laminae limitantes (surrounding *) are visible in representative micrographs.

An interdiffusion zone between enamel and luting agent was found for all luting agents in demineralized and deproteinized specimens that was not visible in polished specimens (Figure 7). In demineralized and deproteinized specimens, tag-like structures were clearly visible and appeared most frequently for PAN specimens (Figure 7).

## 4. Discussion

### 4.1. Discussion of the Method

#### 4.1.1. LV-SEM

In the present study LV-SEM was used to facilitate direct visualization of specimens in their native state as opposed to HV-SEM on replicas or dried and sputter coated specimens [24]. LV-SEM in combination with low voltage (<5 kV) as used in this study is described as a favorable method to show structures of the interface between dentin and restoration materials with less likelihood of artefacts due to desiccation or charge build-up [17,18,25,26]. Using LV-SEM, the native material-contrast from the specimen surfaces can be visualized, because sputter-coating for increasing the surface conductibility is not needed. In this study it was possible to depict the characteristic adhesive interface micromorphology between the luting cement and enamel or dentin in terms of interdiffusion zones, tag-like structures and marginal gaps. In particular, the material contrast available in LV-SEM allowed visualization of interdiffusion zones for CRC+SE and SARCs.

#### 4.1.2. Smear Layer Creation

For creation of a smear layer, wet-abrading by hand (ANSI/CAMI 600) in circular motion was used as it is described as a standard method for mimicking clinical use of rotating diamond burs for finishing purposes (approximately 13 µm grain size) and creating a homogenous smear layer [10,11,16]. A thicker smear layer as created using carbide burs or sand paper of higher grit size for smear layer creation is associated with lower shear bond strength of self-etching and total-etch bonding systems [27]. As finishing procedures are usually performed before application of SARCS or CRC+SE during the preparation and cementation procedures of indirect restorations in a clinical situation, a smear layer of reduced median thickness of 620 nm was created in this study. Likewise, it has also been described by Pashley et al. measuring a smear-layer between 100 and 1000 nm [9]. 

#### 4.1.3. Seating Pressure

It is known that self-adhesive resin cements show better adhesion when a restoration (e.g., a full or partial crown) is seated under pressure compared to restorations seated without pressure [21]. Studies on SARCs have often been criticized for poor results when SARCs were used without application of pressure [21,28]. Therefore, weight was applied during the polymerization process in order to simulate the seating pressure as in clinical cementation procedures [29,30,31], and as recommended for SARCs [32].

### 4.2. Discussion of the Results

Although LV-SEM leads to fewer artefacts due to desiccation than high vacuum SEM [25], compromised specimens for both SARCs due to shrinkage of dentin in the SEM chamber were still found. Some specimens showed complete loss of retention when exposed to vacuum conditions and had to be excluded from semiquantitative examination in dentin. This led to a varying sample size for statistical analyses between groups for all criteria examined in dentin (Figure 6). There were no such desiccation artefacts in enamel. In the present study, marginal gaps along the adhesive interface representing areas of local retention loss between luting agents and enamel were found for SARCs, but not for PAN. Noteworthy, in experiment 2 an interdiffusion zone in terms of a distinct layer between luting agent and enamel was only found in demineralized and deproteinized specimens and only for SARCs, not for PAN (Figure 7). It is discussed in the literature that selective phosphoric acid etching of enamel may be beneficial for enamel adhesion of SARCs due to higher bond-strength to etched enamel resulting from an increased surface energy and a higher amount of micro-porosities in enamel [33,34]. However, SARCs are designed to obtain a stable bond as described for CRC+SE materials with easier and quicker application and less drying of the dentin, which might impair the performance of SARCs [35]. Hence, no additional pretreatment steps such as selective enamel etching were performed. It is worth mentioning that the performance of the luting agents in enamel might be influenced by the direction and density of the cut enamel prisms. This cannot be reliably controlled in molars and may also vary considerably between different enamel areas of the same tooth [36].

In contrast to other in vitro studies characterizing the adhesive interface micromorphology SARCs and dentin or enamel [22,30,37], in the present study morphological interactions between luting agents and dentin were detected, even though the smear layer was not removed or altered before application of the luting agents. Tag-like structures or micromorphological interactions in terms of interdiffusion zones were found, although the dentinal tubules were occluded with smear plugs before material application, which was observed in fractured specimens without application of luting agent (Figure 4). The smear plugs may hinder tag formation.

The demineralized and hybridized dentin surface infiltrated with resin can be visible as a hybrid layer or better termed a resin-dentin interdiffusion zone. For SARCs, the term “hybrid layer” is debatable, because the thickness of this zone does not exceed 1 μm [20,38] and it is difficult to prove that it contains both a collagen network as well as a polymer matrix. Therefore, the terms most frequently used for the zone of interaction between SARCs and dental tissues are “nanohybrid layer” or “interdiffusion zone” [20,36]. In the present study, the term interdiffusion zone was used for all zones of interference between dental hard tissues and luting agents.

The thickness of this interdiffusion zone can reach 4–5 μm for etch-and-rinse and 2–3 μm or less for self-etch adhesive systems, depending on the instrument used for preparation [11]. In the present study, interdiffusion zones below 1 µm instead of a distinct hybrid layer were observed. Ultra-mild self-etch adhesives such as those used in the present study may show lower bond strength and a different morphology when used on thicker smear layers [15].

In the present study, PAN specimens showed the highest amount of characteristic micromorphological interactions of all groups irrespective of the curing mode. The tag-like structures found here might consist of adhesive-resin (ED Primer) reinforced smear plugs and may therefore be less mechanically stable compared to filler-containing tag-like structures. Although they are of higher viscosity, both SARCs formed an interdiffusion zone, but hardly any tag-like structures. The identified tag-like structures resulting from SARCs showed variations such as hollow tag-like structures (Figure 7). Overall, the results indicate that SARCs interact with the smear layer, but do not explicitly dissolve this layer.

Their capability to interact with the underlaying dentin seems to be limited. In experiment 2 of the present study investigating demineralized and deproteinized specimens, it could be observed that some tubule walls were dissolved during demineralization and deproteinization and only brittle, tubule-covering-sheaths remained which may alternatively represent the so-called lamina limitans, in other words, the inner sheath of the peritubular dentin matrix consisting of glucosaminoglycans [39]. The differentiation of the tag-like structures from laminae limitantes (Figure 7) in demineralized and deproteinized specimens, for example, by using energy dispersive X-ray spectroscopy (EDX), might be in the scope of further studies.

In the present study, the luting agents were allowed to set either in DC or SC mode. Another in vitro study investigated a SARC and a conventional resin cement by micro-CT and showed significantly higher polymerization shrinkage when light was applied compared to SC mode [40]. This might be caused by the faster conversion of monomers into polymers. The faster conversion might also lead to less formation of mechanical interlocking as seen in the present study for DC specimens: SARCs tended to have higher incidences of tag-like structures and interdiffusion zones in SC mode.

## 5. Conclusions

Not in enamel, but in dentin, less of the typical micromorphological interactions at the adhesive interfaces were observed for self-adhesive resin cements as compared to the conventional resin cement PAN used with a separate self-etch adhesive. Nevertheless, the first part of the null-hypothesis of the study could not be rejected fully, as micromorphological interactions indicating a mechanical interlocking, in particular interdiffusion zones and tag-like structures, were detected for both self-adhesive resin cements and the conventional resin cement with a separate self-etch step. However, particularly the formation of resin tags was far less pronounced with both self-adhesive resin cements as compared to the conventional resin cement that was used with a beforehand-applied self-etch adhesive. 

The second part of the null-hypothesis could not be rejected, as no significant influence of dual-curing or self-curing on the micromorphology at the adhesive interface was observed. For each material, a tendency for interdiffusion zone and tag-like structures to be found in more specimens after SC compared to DC without statistical significance (*p* > 0.05) was observed.

This might be important for clinical long-term stability of the interface between self-adhesive resin cements and dental hard tissues.

## Figures and Tables

**Table 1:** Luting agents and ingredients according to the manufacturers. The order of ingredients does not represent the respective ingredient concentrations [19].

Material	LOT-No.	Ingredients
Panavia F 2.0 (PAN)	041304	**PAN Paste A:** 10-methacryloyloxydecyl dihydrogen phosphate, hydrophobic aromatic dimethacrylate, hydrophobic aliphatic dimethacrylate, hydrophilic aliphatic dimethacrylate, silanized glass powder, silanated colloidal silica, di-camphorquinone, catalysts, initiators **PAN Paste B:** hydrophobic aromatic dimethacrylate, hydrophobic aliphatic dimethacrylate, hydrophilic aliphatic dimethacrylate, Silanated barium glass filler, catalysts, accelerators, pigments, sodium fluoride **ED Primer Liquid A:** 2-hydroxyethyl methacrylate, 10-methacryloyloxydecyl dihydrogen phosphate, N-methacryloyl-5-aminosalicylic acid, water, accelerators **ED Primer Liquid B:** N-methacryloyl-5-aminosalicylic acid, water, catalysts, accelerators
Clearfil SA Cement (CSA)	023AAB	Silanated barium glass filler, silanated colloidal silica, hydrophilic aliphatic dimethacrylates, triethylene glycol dimethacrylate, bisphenol A-glycidyl methacrylate, catalysts, initiators, di-camphorquinone, 10-methacryloyloxydecyl dihydrogen phosphate, pigments
RelyX Unicem 2 (RXU2)	404603	Silanized glass powder, silane treated silica, 1,12-dodecanedimethacrylate substituted dimethacrylate, methacrylated aliphatic amine, 2,6-di-tert-butyl-p-cresol, sodium P-toluenesulfinate, calcium hydroxyde, barbituric acid derivate, titanium oxide, sodium fluoride

## Data Availability

All relevant data are within the paper files.
